# Near-Infrared Spectroscopy in Extremely Preterm Infants

**DOI:** 10.3389/fped.2020.624113

**Published:** 2021-01-21

**Authors:** Leeann R. Pavlek, Clifford Mueller, Maria R. Jebbia, Matthew J. Kielt, Omid Fathi

**Affiliations:** Division of Neonatology, Nationwide Children's Hospital, Columbus, OH, United States

**Keywords:** neonate, ELGAN, ELBW, NIRS, hemodynamics, prematurity

## Abstract

With advances in neonatal care, survival of premature infants at the limits of viability has improved significantly. Despite these improvement in mortality, infants born at 22–24 weeks gestation are at a very high risk for short- and long-term morbidities associated with prematurity. Many of these diseases have been attributed to abnormalities of tissue oxygenation and perfusion. Near-infrared spectroscopy utilizes the unique absorption properties of oxyhemoglobin and deoxyhemoglobin to provide an assessment of regional tissue oxygen saturation, which can be used to calculate the fractional tissue oxygen extraction. This allows for a non-invasive way to monitor tissue oxygen consumption and enables targeted hemodynamic management. This mini-review provides a brief and complete overview of the background and physiology of near-infrared spectroscopy, practical use in extremely preterm infants, and potential applications in the neonatal intensive care unit. In this mini-review, we aim to summarize the three primary application sites for near-infrared spectroscopy, disease-specific indications, and available literature regarding use in extremely preterm infants.

## Introduction

### History, Physics, and Physiology of Near-Infrared Spectroscopy

Near-infrared energy exists in the 700–1,000 nm wavelength spectrum and was first discovered by the German astronomer (and composer) William Herschel in the 19th century (1). Jobsis et al. applied the principles of near-infrared spectroscopy (NIRS) to medical research in the 1970s and demonstrated that NIRS could monitor regional cerebral blood flow because light wavelengths in the near-infrared range are uniquely suitable to measuring conformational changes in hemoglobin (2, 3). Deoxyhemoglobin exists in a tense, low-oxygen affinity state. Upon binding of one oxygen molecule to a heme subunit, the hemoglobin molecule undergoes a conformational change that results in a more relaxed conformation that increases the affinity of the remaining subunits for oxygen, which forms the basis for the hemoglobin-oxygen dissociation curve (4, 5).

The conformations of deoxy- and oxyhemoglobin display distinct spectroscopic properties and absorption spectra (6). NIRS sensors measure the absorbance of light at wavelengths 700/850 nm, where the near-infrared absorption spectra are maximally separated between oxyhemoglobin and deoxyhemoglobin (7). The NIRS computer calculates the concentrations of deoxyhemoglobin and oxyhemoglobin using the modified Lambert-Beer law and presents the regional oxygen saturation (rSO_2_) as a ratio of oxyhemoglobin/(deoxyhemoglobin + oxyhemoglobin) (8, 9).

NIRS probes generally consist of a single light source (emitting light at two infrared wavelengths) and two down-stream photoreceptors, which measure light absorbance at different tissue depths (10). Absorption of near-infrared light is relatively low and has been detected at depths of several centimeters of biologic tissue (11). The light not detected upon return to the photodetector accounts for light absorbed by the deoxyhemoglobin and oxyhemoglobin. The NIRS computer calculates a tissue-specific rSO_2_ at a tissue depth of 1–2 cm for the entire microcirculation. rSO_2_ is reported as a weighted average of the ratio and therefore, approximately 75–85% of the rSO_2_ reflects the ratio of oxyhemoglobin to total hemoglobin in tissue-specific venules (12). When combined with arterial oxygen saturation (SaO_2_), rSO_2_ can be used to determine the local balance of oxygen delivery and consumption, similar to the mixed venous saturation (SvO_2_). rSO_2_ can be used to calculate the tissue fractional tissue oxygen extraction (FTOE), using the formula FTOE = (SaO_2_ – rSO_2_)/SaO_2_ (13, 14). rSO_2_ has been shown to correlate with SvO_2_ in pediatric cardiac surgery patients (13). Unlike SvO_2_, which requires central venous monitoring to measure, rSO_2_ can be measured non-invasively using NIRS probes.

### Practical Considerations

In neonates, NIRS probes are used to monitor cerebral rSO_2_ (crSO_2_), renal rSO2 (rrSO_2_), and splanchnic rSO_2_ (srSO_2_). NIRS probes for crSO_2_ monitoring are placed midline on the forehead to monitor the frontal lobe gray matter (15, 16). Renal NIRS probes are placed on the back, between the costal margin and iliac crest with the sensor lateral to the spine and reader tip wrapping around the flank (17). Splanchnic NIRS probes are placed over the liver or infra-umbilically (15). Though specific values vary across various NIRS devices (18–20), crSO_2_ is generally lower than rrSO_2_/srSO_2_ because the brain has greater metabolic activity and consumes more oxygen (15). Although adult reports suggest that applied light intensities are not harmful even with prolonged use (10), skin burns, pressure sores, and skin irritation resulting in persistent skin marks at term have been observed in studies of cerebral NIRS monitoring in very preterm infants (21). This review provides a summary (Table 1) of the potential applications of NIRS in the neonatal intensive care unit (NICU).

**Table 1:** Details of studies involving neonates included in this review article.

**Authors (references)**	**NIRS Site**	**Patients included**	**Time of NIRS measurement**	**Number of patients**
Hyttel-Sorensen et al. (21)	Cerebral	<28 weeks	First 72 h of life	166
Hyttel-Sorensen et al. (22)	Cerebral	24/0–27/6 weeks	First 72 h of life	10
Plomgaard et al. (23)	Cerebral	<28 weeks	First 72 h of life	166
Poon et al. (24)	Cerebral, renal	<1,500 g at birth	From diagnosis of hsPDA through last medication dose or surgical ligation	29
Arman et al. (25)	Cerebral, renal, splanchnic	<32 weeks	60 min before pharmacologic treatment of hsPDA and after treatment	31
Montaldo et al. (26)	Cerebral, renal, splanchnic	Full term	First 9 h of life	61
Richter et al. (27)	Cerebral, renal, splanchnic	≤32 weeks	First 5 days of life	80
Bailey et al. (28)	Cerebral, renal, splanchnic	Full term	First 2 days of life	41
McNeill et al. (15)	Cerebral, renal, splanchnic	24–36 weeks	First 21 days of life	14
Terstappen et al. (29)	Renal	<32 weeks	First 72 h of life	16
Bonsante et al. (30)	Cerebral and renal	<32 weeks	First day of life	128
Said et al. (31)	Splanchnic	24–40 weeks	First 14 days of life	40
Gillam-Krakauer et al. (32)	Splanchnic	23/1–30/6 weeks	72 h of monitoring starting in first 14 days of life	25
Cortez et al. (33)	Splanchnic	≤30 weeks	First 14 days of life	21
Dani et al. (34)	Splanchnic	25–31 weeks	60 min before, 30 min after, and 120 min after a bolus feed	54
Dani et al. (35)	Splanchnic	<32 weeks	30 min before a feed to 30 min after	24
Corvaglia et al. (36)	Cerebral and splanchnic	<33 weeks	Monitored during feeding	30
Patel et al. (37)	Splanchnic	<32 weeks	First week of life	100
Fortune et al. (38)	Cerebral and splanchnic	Any gestational age	At admission or immediately prior to surgery, and then daily until discharge	40
Schat et al. (39)	Cerebral and splanchnic	Preterm	48 h after diagnosis of necrotizing enterocolitis	19
Schat et al. (40)	Cerebral and splanchnic	Preterm	48 h after diagnosis of necrotizing enterocolitis	33
Bailey et al. (41)	Cerebral and splanchnic	23/0–36/6	20 min prior to blood transfusion through 12 h after transfusion	35
Banerjee et al. (42)	Splanchnic	Preterm	30–60 min before transfusion and 30–60 min after	59
Banerjee et al. (43)	Splanchnic	<28 weeks	15–20 min before blood transfusion to 15–20 min after	20
Schindler et al. (44)	Splanchnic	23–32	1 h before to 24 h after blood transfusion	41
Marin et al. (45)	Splanchnic	<33 weeks	Time of blood transfusion to 48 h after	17
Marin et al. (46)	Cerebral and splanchnic	24–29 weeks	Time of blood transfusion to 48 h after	8

*NIRS, near-infrared spectroscopy; hsPDA, hemodynamically significant patent ductus arteriosus*.

## Cerebral NIRS

### Background

The survival of extremely low birth weight infants has consistently improved over the past few decades. In addition to improving survival, optimizing neurodevelopmental outcomes is a key area of focus for all institutions that care for such vulnerable infants, especially since one review found that nearly half of surviving infants at <25 weeks had significant short- and long-term neurodevelopmental impairments (47).

Using NIRS in this patient population may help to detect or potentially ameliorate cerebral insults related to regional perfusion and ischemia, primarily intraventricular hemorrhage (IVH) and white matter injury (WMI) (48). Due to a poorly developed cerebral autoregulation system, premature infants are unable to adequately provide consistent cerebral blood flow in the face of stressors (49). NIRS can be helpful to continuously monitor regional brain tissue perfusion and provide objective data about the balance between oxygen delivery and demand. These values have previously been validated against jugular venous saturations in cohorts of neonates (50). While normative values continue to be an area of active research, one large multicenter study has published reference curves for the first 72 h of life in preterm infants (51).

A Cochrane review in 2017 found only one randomized clinical trial looking at the use of cerebral NIRS in preterm infants. While their conclusion did not demonstrate any positive effects of the use of NIRS in preventing brain injury, this study was only powered to detect differences in cerebral oxygenation with the use of NIRS (21). In fact, one of the earliest feasibility studies (SafeBoosC) demonstrated the ability to use NIRS in extremely premature infants and to maintain preterm infants within a predetermined crSO_2_ range (22). In the SafeBoosC-II study, subjects in the NIRS group were successfully maintained in the target crSO_2_ range as compared to a control group. While again not powered to detect clinical differences, there was an association between decreased levels of hypoxia and a lower incidence of severe IVH (23). The SafeBoosC investigators are currently conducting a phase-III follow-up study (SafeBoosC-III, www.safeboosc.eu) to detect differences in clinical outcomes with respect to continuous crSO_2_ monitoring in extremely preterm infants.

### Intraventricular Hemorrhage

IVH remains an important cause of morbidity in mortality in extremely preterm infants (52). While multifactorial, much of the pathophysiology of the development of IVH is tied to the immature vasculature of the germinal matrix (53). It is thought that this impaired cerebral autoregulation is challenged by hypo- and hypercapnia, as well as episodic hypotension that is often compounded by the use of vasoactive agents; a common pathway here is the “pressure-passive” perfusion of the neonatal brain (54). As acute fluctuations in PaCO_2_ are associated with swings in cerebral blood flow, the use of NIRS in mechanically ventilated extremely preterm infants may help to detect and correct these changes before injury can occur (55).

Cerebral blood flow is intimately linked to blood pressure and crSO_2_ patterns passively change with the mean arterial pressure (56). As both extremes of blood pressure have important implications in the development of IVH, crSO_2_ monitoring may help identify such infants and facilitate judicious use of inotropes. One author has suggested using NIRS to minimize fluctuations in cerebral perfusion simply by monitoring those infants who cannot tolerate handling and may benefit from sedation or gentle containment maneuvers (55). Results of studies examining the significance of crSO_2_ and cerebral FTOE values with respect to the development of IVH have varied, as some have demonstrated that an increase in crSO_2_ and a decrease in cerebral FTOE precede severe IVH, while others have seen that these findings are a result of the severe IVH (48). While further research is required to elucidate the true prognostic value, trends in cerebral hemodynamics as measured in crSO_2_ and cerebral FTOE can potentially help detect regional perfusion changes that contribute to the pathophysiology of IVH.

### White Matter Injury

Severe WMI is another significant contributor to poor long-term neurodevelopmental outcomes in extremely premature infants (57). NIRS is being studied and implemented to monitor for patterns of cerebral hypoxia and hypoperfusion that contribute to the development of WMI. The most recognized causes of hemodynamic and vascular disturbances to the fragile preterm brain include hypo- and hypercapnia, left-to-right shunting *via* a hemodynamically significant patent ductus arteriosus (hsPDA), hypoglycemia, anemia, and cortical thinning from post-hemorrhagic hydrocephalus (55). Changes in PaCO_2_ and hemodynamic fluctuations from a hsPDA are perhaps the two most studied etiologies. There are well-described associations of low PaCO_2_ and the development of WMI and cerebral palsy (58). Similar negative impacts are seen with anemia (59) and hypoglycemia (60).

### Patent Ductus Arteriosus

Management of an hsPDA remains a challenging subject in neonatology. With a significant left-to-right shunt, impaired cardiac output can result in end-organ dysfunction. It is speculated that preterm infants with an hsPDA are exposed to decreased cerebral perfusion putting them at increased risk for brain injury (61, 62). While the exact crSO_2_ values during NIRS monitoring are a subject of ongoing research, it is postulated that values below 40–45% for as little as 30 min can have deleterious effects on cerebral blood flow and cerebellar growth (55). The application of NIRS to a preterm infant with an hsPDA may help guide management decisions about ductal closure. Recent prospective studies have shown that crSO_2_ and cerebral FTOE values can be trended during medical or surgical treatment of an hsPDA to help ascertain response (24, 25).

## Renal NIRS

### Background

While use of renal NIRS in the NICU has primarily focused on full-term infants, there is emerging data and evidence for use in premature neonates, including the extremely preterm population. Estimations of normal values for rrSO_2_ in the first days of life in the term neonate have emerged from studies such as that done by Montaldo et al., which investigated renal NIRS values in term neonates starting in the first 15 min of life (26). Such values do not yet exist for premature infants, though some smaller studies examining this question have been published. In 2016 Richter et al. monitored a cohort of 80 infants ≤32 weeks gestation with renal NIRS for the first five postnatal days and found that exposure to maternal antihypertensive medications did not affect regional oxygenation and hemodynamics (27). rrSO_2_ in premature neonates has been observed to be lower than in term neonates within the first 48 h of life (26–28). In 2011 McNeil et al. sought to characterize normal neonatal rrSO_2_ using a cohort of 12 preterm infants 29–33 weeks gestation; the authors observed that rrSO_2_ starts in the mid-80s in the first week of life and decreases over time to mid-60s by the third week of life (15). Terstappen et al. demonstrated that rrSO_2_ was higher overall in growth restricted premature neonates in the first 72 h, compared with control neonates. During the first 72 h of life the rrSO_2_ of the growth restricted group decreased while those of the control group increased (29).

### Acute Kidney Injury

One major potential future application of renal NIRS in the NICU is to monitor for and potentially prevent the development of acute kidney injury (AKI). In 2019 Bonsante et al. became the first to demonstrate the correlation between rrSO_2_ within the first 24 h of life and the future development of AKI in neonates <32 weeks gestation. Using renal NIRS in a cohort of 128 preterm infants, rrSO_2_ was monitored continuously for the first 24 h of life and serum creatinine was monitored daily for seven days. Low rrSO_2_ demonstrated a statistically significant association with the development of AKI (30). Until recently neonatal AKI has largely been an under-recognized disease with significant associated morbidity including long-term development of chronic kidney disease, and an incidence ranging 18–56% in the extremely low birth weight population (63–65). Elevated serum creatinine is the current gold standard for diagnosing AKI, but this takes at least 12–48 h to rise, at which point injury may be irreversible (66). Utilizing renal NIRS to monitor rrSO_2_ in neonates at-risk for AKI could potentially allow for earlier detection and intervention.

## Splanchnic NIRS

### Background

A third major area of potential use for NIRS in extremely premature infants is splanchnic NIRS to monitor gut oxygenation. There are limited studies validating the use of splanchnic NIRS in the NICU. In neonates undergoing surgical correction of congenital heart disease, srSO_2_ correlated with other established measures of oxygenation and perfusion, including lactate, gastric pH, and SvO_2_ (67). Said et al. compared srSO_2_ values with pulse oximetry and umbilical venous catheter oxygen saturations and showed good correlation between NIRS and reference value measurements (31). In stable preterm infants in the first 3 days of life, changes in srSO_2_ values in association with feeds were shown to correlate with changes in superior mesenteric artery blood flow velocity (32). Studies of healthy preterm neonates have shown a decrease in median srSO_2_ over the first week of life, followed by a subsequent increase (15, 33).

### Monitoring of Feeds

NIRS have been utilized to study the response to various types feeding regimens in the preterm infant population, including breast milk compared to formula and bolus compared to continuous feeds (34–36). Dani et al. studied the impact of maternal breast milk, fortified human milk, and preterm infant formula on srSO_2_ at 30 and 120 min after initiation of a bolus feed in 25–31-week infants. srSO_2_ persistently decreased in the preterm formula group, unlike in the two human milk groups, suggesting that formula feeding leads to higher splanchnic FTOE than human milk (34).

A cross-over study in 2013 compared the effect of continuous vs. bolus feedings between appropriate for gestational age and small for gestational age infants at <32 weeks. srSO_2_ increased 30 min after a bolus feed in both groups, while splanchnic FTOE remained stable. On the other hand, srSO_2_ and splanchnic FTOE did not change during a continuous feed in either group of infants, suggesting that energy expenditure is lower during a continuous feed. During both bolus and continuous feeds, srSO_2_ is higher and FTOE is lower in the appropriate for gestational age cohort (35). Conversely, a cross-over study of preterm infants with a median gestational age of 30.1 weeks by Corvaglia et al. revealed increased srSO_2_ beginning at about 1 h after a bolus feed, whereas there was a decrease during continuous feedings (36). Unfortunately, none of these studies focus specifically on the most premature infants between 22 and 24 weeks at birth, and in fact, many limited enrollment to those infants born at >25–26 weeks.

### Necrotizing Enterocolitis

Studies suggest that splanchnic NIRS may play a role in monitoring the hemodynamic changes associated with the development and progression of necrotizing enterocolitis (NEC). Using a cohort of 100 preterm infants <32 weeks gestation, Patel et al. showed that the mean srSO_2_ was lower in the first week of life in the neonates who later developed NEC, and these patients also had significantly more variation in srSO_2_ around feedings in the first 2 weeks (37). Additionally, clinical prediction models utilizing the cerebro-splanchnic oxygen ratio, which compares the srSO_2_ to crSO_2_ (38), and various biomarkers including plasmatic intestinal fatty acid-binding protein (39) have been investigated to improve the sensitivity of NEC prediction in preterm infants. In a prospective cohort study, Schat et al. investigated the utility of splanchnic NIRS to predict the course of NEC in preterm infants. The patients with complicated NEC, defined as bowel perforation requiring surgery or death, had significantly lower srSO_2_ with a higher splanchnic FTOE within 24 h after onset of symptoms when compared to the group that had uncomplicated NEC (40).

### Anemia and Blood Transfusions

A final area of interest is the use of splanchnic NIRS in anemic preterm neonates receiving blood transfusions to further understand the association between NEC and blood transfusions. Blood transfusions have been shown to improve srSO_2_ and decrease splanchnic FTOE in extremely preterm infants (41–43). Changes in srSO_2_ when infants receive feeds during a transfusion have not been consistently shown, with some studies demonstrating no change (44) and others showing a decline (45). Very low birth weight infants who developed transfusion-associated NEC have greater variability in srSO_2_ compared to those who did not develop NEC (46). Additional research in this area is needed to further elucidate mechanisms of transfusion-associated NEC and methods of early prediction of high-risk infants and/or prevention.

## Conclusions

The use of NIRS in preventing cerebral injury in extremely preterm infants remains an active area of challenging research, particularly as it pertains to establishing normative values and consensus within neonatology. The use of “pattern recognition” has often been suggested as a stop-gap measure, that is, recognizing trends in crSO_2_ and the underlying physiology that could be driving these changes (55). The many shared etiologies in cerebral injury in extremely preterm infants does allow for some simplification of clinical investigation when the crSO_2_ values begin to deviate from the “norm.”

While much of the research using NIRS in preterm infants has focused on cerebral NIRS, renal and splanchnic NIRS also have emerging data and potential for clinical and research opportunities. Further studies need to be performed to validate the use of NIRS and determine the appropriate use of this technology, but the possibility of having a non-invasive method of monitoring tissue-level hemodynamics and oxygen balance makes this research a worthwhile endeavor. One key consideration in research and clinical application is the specific NIRS device and sensor used, as there are multiple manufacturers and sizes of sensors. One study has shown consistently higher crSO_2_ using a pediatric or neonatal sensor when compared to an adult sensor, so close attention should be paid to the type of sensor used when interpreting absolute value rSO_2_ results provided in a study (68).

Many studies, even those focused on extremely preterm infants, do not include the most immature babies between 22 and 24 weeks gestation. As this is the patient population at the highest risk of morbidities and mortality associated with prematurity, they stand to benefit the most from precise management of hemodynamic status and monitoring for complications. Outcomes-driven research will ultimately determine if the use of NIRS will become mainstream in most institutions, but the use of regional perfusion monitoring to potentially prevent devastating morbidities is exciting and worth the effort.

## Author Contributions

All authors contributed to the conception and design of this manuscript, drafting and critically revising the initial manuscript, and approval of the final version. All authors are accountable for all aspects of the work.

## Conflict of Interest

The authors declare that the research was conducted in the absence of any commercial or financial relationships that could be construed as a potential conflict of interest.
